# Afternoon Calcium and Vitamin D Supplementation in Water: A Targeted Approach to Improve Laying Hen Nutrition

**DOI:** 10.3390/ani15050720

**Published:** 2025-03-03

**Authors:** Nasima Akter, Thi Hiep Dao, Tamsyn M. Crowley, Sukirno, Aamir Nawab, Amy F. Moss

**Affiliations:** 1 School of Environmental and Rural Science, Faculty of Science, Agriculture, Business and Law, University of New England, Armidale, NSW 2351, Australia or shumi.cvasu13@cvasu.ac.bd (N.A.); tdao2@une.edu.au (T.H.D.); sukirno2@myune.edu.au (S.); anawab@myune.edu.au (A.N.); 2 Department of Dairy and Poultry Science, Faculty of Veterinary Medicine, Chattogram Veterinary and Animal Sciences University, Chattogram 4225, Bangladesh; 3 Institute for Mental and Physical Health and Clinical Translation (IMPACT), School of Medicine, Deakin University, Geelong, VIC 3220, Australia; tamsyn.crowley@une.edu.au

**Keywords:** laying hens, Ca precision feeding, AM/PM feeding, water-soluble minerals, egg quality, serum Ca, vitamin D

## Abstract

A modified AM/PM strategy was evaluated in this study by supplementing calcium (Ca) in afternoon/evening (PM) drinking water instead of feed, offering an alternative to supplying two diets. A 10-week trial was conducted with 288 Hy-Line Brown hens (56 weeks old) housed on the floor in 18 pens (16 hens per pen; 9 replicate pens per treatment). The control diet contained 4.5% Ca, while the AM/PM treatment had 4.1% in feed with additional Ca and vitamin D supplemented through PM drinking water, with other nutrients kept constant in both diets. Daily egg production and weekly feed and water consumption were recorded. Hen weight, welfare scores, and egg quality were assessed at weeks 5 and 10. Blood, ileal digesta, and tibia samples were collected (from 4 hens/pen) at week 10 for serum Ca and vitamin D, digestibility, and bone analyses. Liver and kidney weights, as well as keel bone scores, were also recorded at week 10. Hens on the AM/PM diet consumed significantly less water (171.23 vs. 196.85 mL/bird/day; *p* < 0.001) and had a significantly higher serum vitamin D (27.59 vs. 22.64 ng/mL; *p* = 0.014), with a tendency towards elevated serum calcium levels (31.99 vs. 29.74 mg/dL; *p* = 0.089) compared to the control group. No differences in laying performance, co-efficient of variance (CV%) in individual hen and egg weight, liver and kidney weight, or keel and tibia characteristics were observed between treatments (*p* > 0.05). Similarly, there was no treatment effect on egg quality, and welfare parameters at week 5 and 10, except for smaller eggs (57.13 vs. 57.96; *p* = 0.015), and reduced chest (0.96 vs. 1.16; *p* = 0.028) and belly feather damage (1.34 vs. 1.55; *p* = 0.021), respectively, in hens of the AM/PM treatment compared to the control at week 10. The AM/PM treatment improved ileal digestible energy as is (9.00 vs. 8.22; *p* = 0.022) and as dry matter (10.00 vs. 9.06; *p* = 0.014) compared to the control one. Overall, it demonstrated comparable performance to the control, with added benefits of reduced water intake, better feather condition and vitamin D level and energy digestibility.

## Simple Summary

To meet the circadian requirement of laying hens, a modified AM/PM strategy was applied in the present study by calcium (Ca) supplementation in afternoon/evening water instead of feed as providing two different diets could be expensive for practical farming situations. Therefore, the present trial was carried out by boosting the Ca level in the afternoon/evening via the supplementation of liquid minerals to the drinking water. In this experiment, the performance of hens in the AM/PM treatment was not significantly different to that of hens in the control one. However, other benefits were observed, such as reduced water consumption, leading to cleaner egg production, increased serum Ca and vitamin D levels, better ileal energy digestibility and less evidence of feather damage, indicating a better welfare for hens offered by the AM/PM treatment.

## 1. Introduction

Calcium (Ca) is a critical nutrient in the diet of laying hens, primarily due to its role in eggshell formation, skeletal health, and overall productivity. About 94–97% of an eggshell is composed of calcium carbonate [1], and a significant portion of this Ca is derived directly from the hen’s diet [2]. An insufficient supply of dietary Ca can lead to poor eggshell quality, increased incidences of cracked or soft-shelled eggs, and depletion of skeletal Ca reserves, potentially resulting in osteoporosis or fractures [3,4]. Furthermore, Ca interacts with vitamin D to enhance absorption and utilization. Vitamin D plays a vital role in Ca absorption in laying hens by enhancing the uptake of Ca in the intestines through the stimulation of calcium-binding proteins [5,6,7]. This process is especially important for eggshell formation, which requires high levels of Ca, particularly during the evening/night. Sufficient vitamin D ensures a consistent supply of Ca in the bloodstream, supporting strong eggshells and minimizing the risk of breakage [8]. Moreover, vitamin D regulates the balance of Ca and phosphorus, preserving bone health and preventing skeletal depletion [9]. This is especially important when hens are raised indoors, as they have limited exposure to sunlight, which is necessary for the natural synthesis of vitamin D [10]. Since Ca and vitamin D are complementary and interdependent for effective utilization, a deficiency in either can disrupt Ca metabolism, leading to negative impacts on egg production and shell quality [11,12]. Adequate Ca intake supports not only eggshell formation but also metabolic and physiological functions. For an optimal performance, hens require approximately 4–5% Ca in their diet during peak production [13]. The timing of Ca supplementation also plays a crucial role. Hens utilize dietary Ca more efficiently for shell formation during the evening and night, aligning with the timing of eggshell deposition [14]. This has led to strategies like the AM/PM feeding approach to ensure sufficient Ca availability during critical periods.

AM/PM feeding has recently garnered significant attention from poultry nutritionists, particularly concerning the precise nutritional requirements of laying hens. Precision laying hen feeding aims to provide nutrients in optimal amounts and timing, minimizing waste and maximizing efficiency [15,16]. The AM/PM feeding strategy is designed to meet the specific nutritional requirements of laying hens by aligning nutrient delivery with their circadian biological processes. This concept emphasizes providing different nutrient compositions in the morning (AM) and evening/night (PM) to address the hen’s physiological needs during egg formation [17,18,19], particularly eggshell deposition, which occurs predominantly during the night [16]. The AM/PM feeding strategy embodies this principle by synchronizing Ca delivery with the hen’s physiological demand for shell formation [20,21,22]. Since the amount and timing of calcium supplementation are crucial for laying hens, providing an afternoon diet lacking sufficient calcium can negatively impact eggshell quality, deplete skeletal Ca, leading to poor bone health, reduce productivity, and adversely affect their welfare [3,4].

Traditional laying hen diets provide an average percentage of Ca throughout the day despite the higher physiological demand in the afternoon or evening by hens, which can cause Ca deficiency and related issues [16]. Additionally, unnecessarily high levels of dietary Ca in the morning can interfere with the metabolism of other nutrients [23,24,25]. This imbalance not only disrupts nutrient utilization [24,25] but also results in the wastage of Ca, ultimately lowering feed efficiency and increasing production costs. To address this challenge, poultry scientists are now exploring various AM/PM or split feeding strategies designed to align more effectively with the cyclic nutritional demands of laying hens, particularly for Ca and other essential nutrients [18,26,27].

While AM/PM feeding strategies offer several advantages in aligning nutrient supply with the physiological demands of laying hens, there are some limitations associated with their implementation. This feeding regime requires separate feed storage and feed lines on-farm, which can increase farm establishment costs, particularly for large-scale operations. Many existing poultry farms lack the necessary infrastructure, which is the main hesitation in the implementation of AM/PM feeding regimes in practice. Additionally, most of the existing research on AM/PM strategies for laying hens focuses on methods to optimize Ca levels in the diet to meet the hens’ physiological requirements. However, to the best of the authors’ knowledge, there are currently very few reports, like those of Damron and Flunker [28] and Yi et al. [29], available on the efficiency of direct supplementation of Ca in drinking water in laying hen production and none of them address the time of administration as a targeted strategy. This gap highlights an opportunity for further investigation into alternative approaches of AM/PM feeding to efficiently deliver Ca during critical periods. Hence, the present study investigates the effects of supplementing Ca along with vitamin D through afternoon drinking water on laying performance, egg quality, and the welfare of hens compared to the traditional laying hen diet.

## 2. Materials and Methods

This trial was carried out at the Laureldale Free-Range Poultry Research Centre, part of the University of New England, located in Armidale, New South Wales, Australia. The study design received approval from the Animal Ethics Committee of the University of New England (approval number: ARA21-105) and conformed to the guidelines set forth in the Australian Code of Practice for the Care and Use of Animals for Scientific Purposes [30].

### 2.1. Animal Husbandry

**Figure 1:**
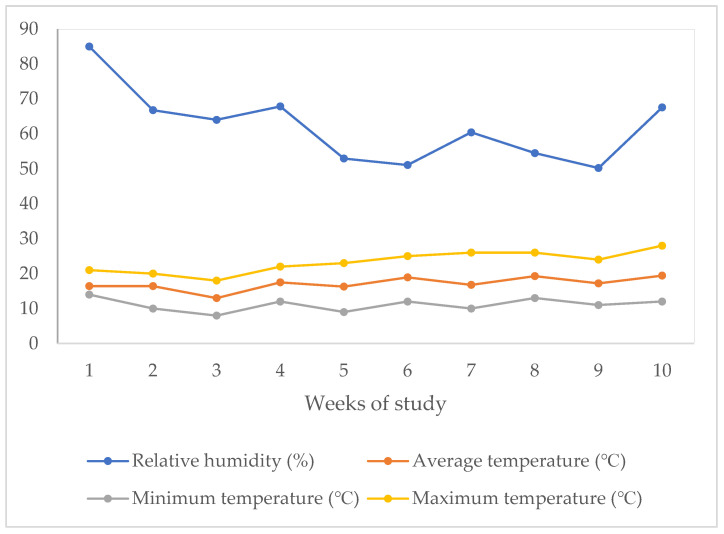
Temperature and relative humidity of the hen house during 10 weeks of study.

A total of 288 Hy-Line Brown laying hens were utilized in this study. Prior to the experiment, the hens participated in two other studies at our facility. For the initial research, pullets were obtained from a commercial layer farm in Tamworth, New South Wales, Australia, at 15 weeks of age (WOA) and reared in cages. At the conclusion of the prior trial, the hens were reared for 3 weeks on a standard commercial laying diet ((Barastoc—Premium Top Layer Mash, containing 16.5% crude protein, 2.5% crude fat, 6% crude fiber, 0.3% salt, 8.0 mg/kg copper, 0.3 mg/kg selenium, and 3.6% calcium; Melbourne, VIC, Australia) as an adaptation period. To prevent any potential carryover effects, we selected healthy hens after each trial by analyzing their bone health and nutrient metabolism. Then, the hens (56 WOA) were randomly assigned to 18 floor pens in a semi-control shed, with 16 birds per pen, resulting in a stocking density of 2.31 hens/m^2^, and there were no significant differences in body weight (average weight 2181 g/hen, *p* > 0.05)) or laying performance (average egg production 94.86%, *p* > 0.05) between the experimental groups. Each pen measured 4.8 m × 1.8 m and was equipped with a round feeder trough (39 cm height × 43.5 cm diameter × 1.36 m circumference), an automatic nipple drinker system (nipples set at bird eye height), a three-rung perch (1.07 m length × 64 cm width × 80 cm height), and a roll-away nest box (34 cm length × 29 cm width × 24 cm height). The pens adhered to the standards outlined in the Australian Model Code of Practice for the Welfare of Animals: Domestic Poultry [31]. Fresh wood shavings (5–7 cm deep) were used as bedding, and pens were separated by wire panels with shade cloth (1 m high) to provide visual isolation. The experiment began when the hens were of 56 WOA, with birds allocated to two dietary treatments. Feed and water were supplied ad libitum throughout the trial. Lighting was provided via specialized poultry white LED bulbs (IP65 Dimmable LED Bulb, B-E27:10W, 5K; Eco Industrial Supplies, Zhenjiang, China), set to a 16 h light and 8 h dark cycle, turning on at 4:00 h and off at 20:00 h. Temperature and relative humidity inside the shed were recorded daily in the morning and evening using a thermometer/hygrometer (Temp Alert, FCC RoHS, 2011/65/EU, FCC: R17HE910, S4GEM35XB, Boston, MA, USA). Figure 1 shows the average ambient temperature (°C) and relative humidity (%) within the shed from week 1 to week 10 of this study. Throughout this study, the mean air temperature was 17 °C, with a range between 13 °C and 19 °C, while the average relative humidity was 62%, fluctuating between 50% and 85%. The maximum temperature varied from 18 °C to 28 °C, with an average of 23 °C, and the minimum temperature ranged from 8 °C to 14 °C, with an average of 11 °C. The hens were rehomed after the completion of this trial. As all hens in different treatment pens were exposed to the same temperature, we did not consider temperature as a covariate, as any temperature-related effect like feed and water intake would have been consistent across all groups due to the uniform distribution of pens throughout the shed.

### 2.2. Experimental Treatments and Water Supply

This 10-week trial (56–65 WOA) involved two dietary treatments: a control diet and an AM/PM diet. Each treatment included 9 replicate pens with 16 hens per pen, totaling 144 hens per treatment. The control diet was formulated to meet breed-specific requirements with 4.5% Ca, while the AM/PM diet met all requirements except for a reduced Ca level of 4.1%. The detailed composition of these diets is provided in Table 1. Prior to formulating the diets, the main nutrient content of the ingredients was analyzed using near-infrared reflectance spectroscopy (Foss NIR 6500, Hillerød, Denmark), calibrated with Evonik AMINONIR Advanced standards. Both diets were prepared in mash form and analyzed for actual nutrient content using standard analytical methods [32]. The calculated and analyzed nutrient values of the experimental diets are presented in Table 2. For the AM/PM treatment, hens received a Ca and vitamin D supplement through their afternoon (PM) drinking water, while morning (AM) water and all water for the control group remained plain. Calcium lactate was used as the Ca source for drinking water as it is commonly used in liquid Ca supplements and is readily available in bulk from a major Australian chemical supplier (Galaxium Pentahydrate Pearls, Galactic SA, Escanaffles, Belgium). The vitamin D supplement was a commercially available product, Zagrosol AD_3_E (Zagro Australia Pty Ltd., Perth, WA, Australia), administered according to the manufacturer’s recommendations. The PM drinking water contained Ca lactate at a concentration of 3.33 g/100 mL, providing 0.4 g/100 mL of Ca. This dosage was estimated to supply approximately 0.6 g of Ca per hen daily, based on an intake of 150 mL/hen during the PM phase. Water was supplied via separate calibrated barrels (control, AM, or PM barrel) to enable accurate water intake calculations and facilitate the switching of water sources. The water supply was exchanged daily at 8 AM and 4 PM for the AM/PM treatment.

### 2.3. Sampling and Data Recording

Individual hen and egg weight were recorded at weeks 5 and 10 to calculate the co-efficient of variance (CV%) or uniformity. Daily records were kept for egg number and weight, while internal and external egg quality were evaluated at weeks 5 and 10. Feed and water intakes were monitored weekly, with AM and PM water consumption recorded separately. Performance indices such as hen day egg production (HDEP), egg mass, and feed conversion ratio (FCR) were calculated using the following equations:

$$\begin{array}{c} HDEP\left(\%\right)=\frac{Total\;number\;of\;eggs}{Total\;number\;of\;hens\times 7\left(days\right)}\times 100\\ Egg\;mass(g/day)=HDEP(\%)\times Average\;egg\;weight(g)\\ FCR=\frac{kg\;of\;feed\;consumed}{kg\;of\;egg\;mass} \end{array}$$

The welfare scoring of hens was carried out according to the Welfare Quality Network [33] at weeks 5 and 10. At the end of the experiment (week 10), 4 hens/pen (36 birds/treatment) were weighed, stunned and sacrificed humanely to collect blood, liver, kidney, tibia and ileal digesta samples. The weights of the liver and kidney were recorded, while blood samples were collected for serum Ca and vitamin D study. The tibias of hens were sampled to analyze bone characteristics. Ileal digesta samples were collected to determine nutrient digestibility. The sampled hens’ keel bone damage scores were also documented according to the Welfare Quality Network [33] on the same day.

### 2.4. Egg Quality Analysis

To assess the internal and external quality of eggs, 144 eggs (8 eggs per pen, 72 eggs per treatment) were collected in the morning during weeks 5 and 10 and transported to the laboratory. Except for eggshell weight and thickness (which required drying before measurement), all quality parameters were assessed within four hours of collection. Egg length (mm) and width (mm) were measured using a digital Vernier caliper (Kincrome^®^, 0–150 mm scale, Scoresby, VIC, Australia) to calculate the egg shape index (SI = width/length × 100). Eggshell reflectivity refers to the amount of light reflected from the surface of an egg [34], measured using a shell reflectivity meter (Technical Services and Supplies, Dunnington, York, UK), while eggshell breaking strength and internal egg quality parameters were analyzed with a digital egg tester (DET6500^®^, Nabel Co., Ltd., Kyoto, Japan). Yolks were separated from albumen using Whatman filter papers (CAT No. 1541–090, Whatman^®^, Buckinghamshire HP7 9NA, Amersham, UK) and weighed. Albumen weight was calculated by subtracting the yolk and eggshell weights from the total egg weight. Eggshells were cleaned, air-dried for at least 72 h, and weighed using a precision analytical balance (Adventurer TM, Model AX423, Ohaus^®^, Newark, NJ, USA). Thickness, including the outer shell membrane, was measured using a custom-built gauge (Mitutoyo Dial Comparator Gauge, Model 2109-10, Kawasaki, Japan).

### 2.5. Serum Ca and Vitamin D Analysis

On the sampling day (week 10), blood samples were collected from hens by severing the jugular vein into silica-coated vacutainers (Becton, Dickinson UK Limited, Plymouth, UK) containing serum separator polymer gel intended for serum Ca and vitamin D analysis. The samples were promptly transported to the laboratory in a cool box to maintain their integrity. Further processing and analysis of serum Ca were conducted using the method described by Jahan et al. [18], while vitamin D analysis followed the procedure outlined by Rana et al. [35].

### 2.6. Nutrient Digestibility

After collecting ileal digesta samples, they were processed and analyzed for dry matter (DM), energy, and protein (nitrogen) concentrations using the methods described by Jahan et al. [18]. The titanium dioxide (TiO_2_) content in the feed and ileal digesta samples was determined following the procedure reported by Short et al. [36]. Finally, energy and nitrogen digestibility were calculated using following equations described by Jasek et al. [37].

$$IDE=GE_{diet}-\left(GE_{digesta}\times \left(\frac{Ti_{diet}}{Ti_{digesta}}\right)\right)$$

$$IDEC=1-\left(\frac{Ti_{diet}\times GE_{digesta}}{Ti_{digesta}\times GE_{diet}}\right)$$

$$IDNC=1-\left(\frac{Ti_{diet}\times N_{digesta}}{Ti_{digesta}\times N_{diet}}\right)$$

Here, IDE = Ileal digestible energy, IDEC = Ileal digestible energy co-efficient, IDNC = Ileal digestible nitrogen co-efficient, GE_diet_ = Gross energy of diet/feed sample, GE_digesta_ = Gross energy of digesta sample, Ti_diet_ = Titanium dioxide of diet/feed sample, Ti_digesta_ = Titanium dioxide in digesta sample, N_die_t = Nitrogen in diet/feed sample, N_digesta_ = Nitrogen in digesta sample.

### 2.7. Tibia Characteristics

The quality parameters of the tibia samples, including length, breadth, weight (wet and dry), and breaking strength, were assessed as follows. The length (from the tip of the proximal end to the tip of the distal end) and the width (at the midpoint) of the air-dried tibias were measured using a Kincrome 0–150 mm Digital Vernier caliper (Kincrome, Scoresby, VIC, Australia), while bone density was calculated using the Seedor index, defined as the ratio of the air-dried bone weight (mg) to its length (mm), following the method of Seedor et al. [38]. The breaking strength of air-dried tibias was measured using an Instron^®^ electromechanical universal testing machine (Instron^®^ Mechanical Testing Systems, Norwood, MA, USA). The breaking strength was measured with a 3-point flexure test setup, utilizing a 300 kN load cell and a speed of 0.2 mm/second, with data recorded at 20 points per second. The universal material testing software Bluehill (ver.2, Instron^®^Mechanical Testing Systems, 825 University Ave., Norwood, MA, USA) was used for recording the data. The mechanical force was applied at the midpoint of the bone, which was supported at a 2 cm distance between two fixed points (50 mm apart). Following the strength tests, the fractured tibia samples were weighed and placed in crucibles for ashing. The samples were ashed in a muffle furnace (Carbolite, Sheffield, UK), initially at 350 °C for one hour, followed by 600 °C for 16 h. After cooling, the samples were reweighed. The ash content was determined by dividing the ash weight by the oven-dried bone weight and multiplying by 100 to express the result as a percentage (%).

### 2.8. Data Analysis

The collected data were organized using Microsoft Excel and analyzed with IBM SPSS Statistics software (Version 28.0.1.0, IBM Corp., Armonk, NY, USA), setting the significance level at 0.05. Prior to analysis, data were checked for normality and homogeneity of variances across dietary treatments. A one-way ANOVA was performed using univariate General Linear Models (GLMs), with treatment specified as a fixed effect, to evaluate mean differences among the dietary groups. Tukey’s post hoc test was applied to identify pairwise differences when significant effects were detected. *p*-values ≤ 0.05 were considered statistically significant, while values between 0.05 and 0.10 were interpreted as indicative of trends.

## 3. Results

### 3.1. Laying Performance

The overall laying performance from weeks 1 to 10, including the interim performance of weeks 1 to 5 and 6 to 10, is reported in Table 3. There was no significant difference in the performance of the hens offered either the AM/PM or the control diet (*p* > 0.05).

### 3.2. Feed and Water Intake

**Figure 2:**
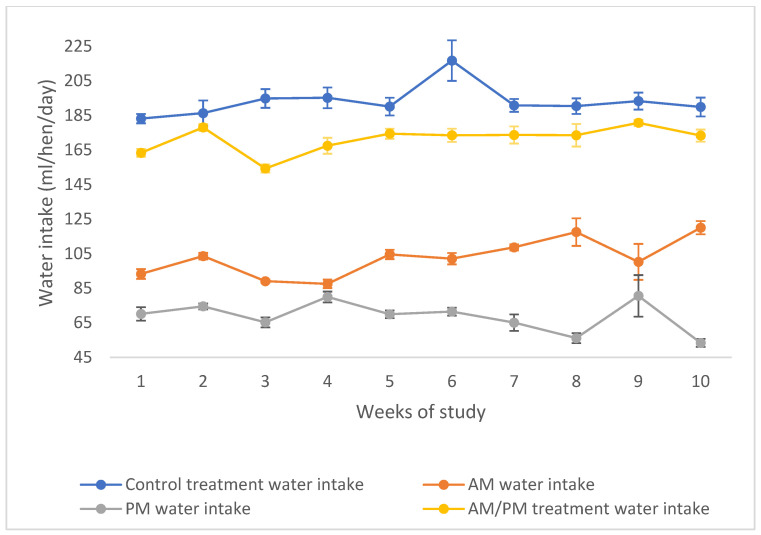
Weekly water intake of control and AM/PM treatments along with individual AM and PM intake from week 1 to 10 of this study. The dot points represent means and error bars present standard errors of the means.

Table 4 presents the feed and water intake of hens from weeks 1 to 10, including the interim intake for weeks 1 to 5 and 6 to 10. Feed intake remained consistent between treatments (*p* > 0.05), while hens of the AM/PM treatment, supplemented with Ca and Vitamin D in PM drinking water, consumed significantly less water compared to control hens (171.23 vs. 196.85 g/bird/day; *p* < 0.001) throughout the entire 10-week trial. There was a lower trend of feed intake in the AM/PM treatment compared to the control one during the whole 10 weeks of the trial (144.41 vs. 152.55 g/bird/day; *p* = 0.083). Figure 2 illustrates the weekly intake of AM and PM water of the AM/PM treatment, along with the average water intake for both the control and AM/PM treatments. The data clearly show that hens in the AM/PM treatment consumed less water overall compared to the control. Additionally, within the AM/PM treatment, the average intake of AM water (102.63 mL/hen/day) was higher than that of PM water (68.60 mL/hen/day) over the 10-week experimental period.

### 3.3. Egg Quality

The external and internal quality of eggs measured at weeks 5 and 10 are stated in Table 5. There were no significant differences in any egg quality parameters between treatments at both weeks, except that eggs from the AM/PM treatment had a significantly higher egg shape index (0.769 vs. 0.759; *p* = 0.021) compared to the control treatment at week 5. Additionally, there was a trend toward lower yolk height in AM/PM eggs at week 10 (*p* = 0.076; Table 5).

### 3.4. Individual Weight of Hen and Egg

The weights of the hen and egg, as well as the co-efficient of variance (CV%) in individual hen and egg weight at weeks 5 and 10, are reported in Table 6. Hen weight and egg weight were found to be consistent across treatments at both weeks 5 and 10 of the trial (*p* > 0.05). Similarly, there was no significant difference in the CV% or uniformity in either the weight of hens or that of eggs between treatments (*p* > 0.05). However, there was a trend toward a higher CV% in individual hen weight for the AM/PM treatment at both week 5 (9.44 vs. 8.04; *p* = 0.067) and 10 (9.88 vs. 8.36; *p* = 0.071) compared to the control. No mortality was observed during this study.

### 3.5. Nutrient Digestibility

Table 7 shows ileal energy and nitrogen (N) digestibility at week 10 of this study (65 WOA in hens). The findings revealed that the ileal energy digestibility (IDE) as dry matter (DM) (10.00 vs. 9.06; *p* = 0.014) was significantly higher in hens receiving the AM/PM treatment compared to those on the control treatment.

### 3.6. Serum Ca and Vitamin D

Serum Ca and vitamin D levels at week 10 are reported in Table 8. Hens offered AM/PM diets and Ca in the water had numerically higher serum Ca levels than hens offered the control diet (31.99 vs. 29.74 mg/dL; *p* > 0.05), while hens of the AM/PM treatment had a significantly greater serum vitamin D level than the control ones (27.59 vs. 22.64 ng/mL; *p* = 0.014).

### 3.7. Kidney Weight, Liver Weight and Keel Bone Anatomy

Table 9 presents the average kidney weight, liver weight, and keel bone damage score of hens at week 10. Dietary treatments did not affect liver or kidney weight, nor were keel bone abnormalities observed in the present study (*p* > 0.05). However, the average kidney weight found was numerically higher in the control treatment (*p* = 0.0814; Table 9).

### 3.8. Tibia Characteristics

Bone characteristics of the tibia were measured at 10 weeks, as outlined in Table 10. There was no effect of treatment on the weight, length, width, or breaking strength of bone (*p* > 0.05). However, hens offered the AM/PM treatment had a significantly lower ash content (as-is %) of bone compared to control hens (39.73% vs. 40.30%; *p* = 0.024).

### 3.9. Hen Welfare Scoring

Hen welfare scoring at weeks 5 and 10 is given in Table 11. There were no effects of dietary treatments on comb wounds, back of head, neck, back, wing, tail, or vent plumage, keel bone, body condition, or footpad lesion scores (*p* > 0.05). However, hens fed the AM/PM treatment showed significantly less damage to their chest feathers (0.91 vs. 1.16; *p* = 0.028) and belly feathers (1.34 vs. 1.55; *p* = 0.021) compared to those on the control diet at week 10. Additionally, there was a decreasing trend in damage to neck (*p* = 0.092) and wing feathers (*p* = 0.078) in the AM/PM group at week 10.

## 4. Discussion

Ca is a key nutrient in laying hen diets, directly influencing egg quality and laying performance. Poultry nutritionists have long focused on optimizing Ca supplementation in hen rations as adequate Ca intake is crucial for hens to sustain consistent egg production [39]. Factors such as Ca levels, particle size, and timing of supplementation have been shown to influence Ca utilization, laying performance, eggshell quality, bone health, and hen welfare at various stages of production [14,26,27,40,41,42,43]. On the other hand, vitamin D is essential in the diet of laying hens as it plays a crucial role in Ca and phosphorus metabolism, which are vital for eggshell formation and bone health. Adequate vitamin D ensures optimal Ca absorption from the gut, supporting strong eggshells and reducing the risk of osteoporosis and fractures [5,6,7]. It also regulates blood Ca levels, preventing hypocalcemia, which can impair egg production and overall health [11,12]. Additionally, vitamin D influences immune function and muscle strength in hens [44]. Therefore, maintaining appropriate vitamin D levels is crucial for maximizing productivity, welfare, and longevity in laying hens. Thus, poultry nutritionists focus on formulating diets with precise Ca and vitamin D supplementation strategies to maximize productivity while ensuring the health and well-being of laying hens. The present study aimed to investigate the effects of waterborne Ca and vitamin D supplementation provided in PM drinking water as a part of a modified AM/PM feeding strategy on laying performance, egg quality, and hen welfare.

The overall performance data of the present study indicated that Ca and vitamin D supplementation in PM drinking water did not significantly influence key production metrics such as egg weight, hen day egg production, egg mass, or feed conversion ratio (FCR) despite the experimental diet having a lower Ca content (4.1%) compared to the control diet (4.5%). These findings align with studies by Damron and Flunker [28] and Yi et al. [29], which found no differences in egg production or egg weight when hens received Ca supplementation in water, though they used a Ca supplement in water throughout the day instead of PM water only. Similarly, An et al. [40] found no changes in egg production or egg mass in hens fed varying levels of dietary Ca during the late laying stage (70–79 WOA). Experiments on Ca supplementation at different times of day by Saki et al. [43], de los Mozos et al. [45], and Faruk et al. [46] also showed no differences in production indices. Jahan et al. [18] also observed comparable egg weight and production in hens fed either a control diet or an AM/PM diet, supporting the present study’s findings. Similarly, Jiang et al. [47] noted consistent egg weights in hens fed varying levels of Ca during the early laying stage (19–27 WOA). Conversely, Qui et al. [48] reported improved laying rate and egg mass with dietary Ca lactate supplementation in aged hens (62–73 WOA), a result that contrasts with the current study. These comparisons suggest that while some studies partially support the findings, differences in age, Ca supplementation strategies, and experimental conditions may influence outcomes.

Feed intake in hens provided with Ca lactate and vitamin D in PM drinking water did not differ significantly from that in the control treatment in the present study, although a decreasing trend in feed intake was noted in the AM/PM treatment. This result partially agrees with Damron and Flunker [28], who observed that waterborne Ca supplementation depressed feed intake. However, other studies reported consistent feed intake across hens given varying dietary Ca levels [40,47]. The feed intake per hen per day in the present study ranges from 142 to 152 g (Table 4), which is around 30–40 g higher than the Hy-Line brown standard daily intake. According to the production guide, the standard daily feed intake for Hy-Line Brown laying hens is approximately 110 g/hen [49]. However, in the current study, the hens consumed 142–152 g/hen/day, which is noticeably higher than the recommended amount. Two possible explanations for this increased feed intake are suggested. First, the lower overnight temperatures and generally cooler days during most of the study period may have prompted the hens to consume more feed to maintain optimal body temperature. Second, the hens had ad libitum access to feed throughout the day, unlike in commercial farms, where feed is typically provided in specific amounts at set times, such as once or twice a day. These factors likely contributed to the higher feed intake observed in this study compared to the Hy-Line laying hen catalog.

Water intake significantly differed between treatments in the current study, with reduced water consumption observed in hens provided with Ca and Vitamin D supplemented PM water. This finding aligns with the work of Damron and Flunker [28], though Yi et al. [29] found no differences in water intake when hens were given water containing varying levels of Ca and Mg compared to regular tap water. The observed reduction in water intake might be attributed to aversion to the taste of water containing Ca lactate and vitamin D, as Yi et al. [29] used Mg rather than vitamin D alongside Ca. Although a lower water intake is often associated with decreased egg production due to the close relationship between water and feed intake [50], the hens in the AM/PM treatment in the present study did not exhibit reduced feed intake or egg production. This could be because the water intake remained within acceptable limits. Importantly, a reduced water intake may benefit hens raised on the floor by lowering the moisture content of excreta [51,52], which can contribute to cleaner eggs [53], reduced ammonia levels in the shed [54,55], improved litter conditions, and better bird welfare by mitigating footpad dermatitis [56].

In the present study, egg quality scores, both external and internal, were similar between treatments, except for the shape index of eggs at 60 WOA. A reduced shape index (75.9%) was seen in the control eggs compared to the AM/PM eggs (76.9%). However, both shape index scores remained within the standard range (72 to 76%) [57]. Eggs with an optimal shape index are less likely to break during handling, storage, and transport, improving shelf life, reducing economic losses, and meeting consumer preferences [57,58]. By optimizing Ca and vitamin D intake with timing, the AM/PM treatment may improve eggshell formation during the night when shell calcification is at its peak [16]. This could lead to better eggshell strength and uniform deposition, potentially affecting egg shape consistency. However, the shape index is also influenced by genetic factors, age, and overall nutrient intake [59]. Therefore, the observed difference in the shape index between the AM/PM and control treatments in the present study would likely result from a complex interaction of these factors. Further studies specifically examining this relationship are needed for conclusive evidence. Other major quality parameters, such as shell weight, shell thickness, shell breaking strength, and Haugh unit, remained consistent across the treatments in the present study. Yi et al. [29] partially support these findings as they did not observe any effect of Ca-and-vitamin-D-supplemented water on eggshell color, yolk color, Haugh unit, or albumen height. In another study, dietary supplementation of Ca lactate improved eggshell thickness, strength, and albumen height, but had no effect on other parameters, partially supporting the present study’s findings [48]. Studies on afternoon Ca supplementation via the AM/PM feeding regime also showed that egg quality, except for yolk color, was unaffected by the dietary treatments [18,60], which aligns with the majority of the egg quality results in the present study.

Previous AM/PM studies [18,20] reported no difference in hen and egg weights between treatments, which agrees with the present study’s findings. In this study, both hen and egg weights also remained consistent across treatments. Additionally, the CV% for hen and egg weights were similar between treatments, indicating that Ca and vitamin D supplementation through PM drinking water did not negatively affect the uniformity of hen and egg weight. This observation is also supported by Jahan et al. [18].

Ileal digesta samples were analyzed to determine energy and nitrogen (N) digestibility in the present study. The results showed that energy digestibility was significantly higher in hens of the AM/PM treatment compared to the control hens. This suggests that the AM/PM treatment improves the efficiency of energy digestion and absorption of hens. Previous studies indicate that excessive Ca in the diet can significantly reduce nutrient digestibility and feed efficiency in poultry as well as in pigs [23,24,25]. High dietary Ca from limestone tends to raise gut pH because of its acid-binding properties [61], which can lead to the formation of Ca–phytate complexes [62] and may even precipitate with phosphorus [63]. This change in gut pH can disrupt the digestive process and enzyme activity, further impairing nutrient absorption [24] and hindering growth performance [64] in chickens. The adverse effects of excessive Ca on phosphorus utilization, as well as its impact on several other essential nutrients and energy, render the excess of this mineral an antinutrient [63]. In the present study, hens on the AM/PM treatment received lower levels of Ca through dietary limestone compared to the control treatment, which may have facilitated better energy digestion.

The analysis of serum Ca in the present study indicated that Ca levels were not significantly affected by afternoon waterborne Ca and vitamin D supplementation. However, hens in the AM/PM treatment tended to have slightly higher serum Ca levels (*p* = 0.089) than those in the control group. This increase indicates a potential trend toward improved Ca retention in the AM/PM treatment of the present study. The timing of Ca supplementation, along with additional vitamin D in the AM/PM treatment in the present study, might play a role in increasing the serum Ca level [5,6,7]. Other studies have shown varying effects of dietary Ca levels on serum Ca, with some reporting increases [14,47] and others observing no change [40]. These discrepancies may stem from differences in the mode and timing of supplementation, hen strain and age. Ca source or level could be another factor to be considered as Qui et al. [48] found that substituting limestone with 0.5% dietary Ca lactate elevated the serum Ca and P levels. Moreover, in the present study, hens in the AM/PM treatment group had significantly higher serum vitamin D levels compared to those in the control treatment, which is expected, as they received an additional vitamin D supplement alongside calcium lactate through the water. This resulted in a higher total dietary intake of vitamin D (from both feed and PM water) for the AM/PM hens compared to the control group, which only received vitamin D through the feed. This increased intake likely contributed to the significant elevation in serum vitamin D levels observed in the AM/PM hens in this study. Since there is no widely accepted reference range for standard serum vitamin D levels in laying hens, as vitamin D requirements vary depending on several factors such as age, species, breed, diet, stage of production, and environmental conditions, we cannot speculate on the potential long-term effects of higher serum vitamin D levels on hen metabolism. As our study was limited to a short duration of 10 weeks focused on a specific phase of laying hen production, we are unable to draw any conclusions about the long-term impact of elevated serum vitamin D on metabolism. Further investigations are necessary to develop a more comprehensive understanding of these effects.

Kidney and liver weights in laying hens are critical indicators of health, metabolism, and overall physiological status [65,66]. The liver plays a central role in nutrient metabolism, including protein synthesis, fat metabolism, and the storage and release of essential nutrients like vitamins and minerals (Ca), which are crucial for the production of egg albumen, yolk and shell [65,67]. Similarly, the kidneys in laying hens play a vital role in Ca and phosphorus (P) homeostasis [50,68]. In the present study, the liver and kidney weights were within the standard range and did not differ between treatments, suggesting that the experimental diets had no adverse effects on these organs. Based on current research, AM/PM feeding regimens in laying hens are unlikely to significantly affect liver and kidney weight or health but could potentially lead to slight improvements in liver and kidney function by better managing Ca intake throughout the day. This approach could reduce stress on these organs, such as by minimizing excessive Ca accumulation, which can strain the kidneys [69]. This strategy may also support liver heath in laying hens by ensuring sufficient Ca supply when required [70] and promoting efficient Ca absorption through vitamin D supplementation [5,6,7]. However, further research is needed to fully understand the effects of this feeding strategy on organ health.

Keel bone health is a critical aspect of laying hen welfare, productivity, and longevity [71,72,73,74]. The keel bone supports the hen’s skeletal structure and plays a vital role in movements such as perching and wing flapping. Poor keel bone health, including fractures or deformities, can cause pain, reduce mobility, and impair a hen’s ability to access resources like food, water, and nesting areas [75]. Both treatment groups in the present study exhibited minimal keel bone damage, with no significant differences between them (*p* = 0.299). However, there was a downward trend in keel bone damage scores in hens receiving the AM/PM treatment compared to the control group, suggesting its potential to prevent keel bone damage. Providing Ca with vitamin D through afternoon and evening drinking water in the AM/PM treatment may ensure improved Ca utilization, reducing the risk of keel bone fractures or deformities in laying hens in the present study [5,6,7,76]. However, keel bone health is also influenced by other factors, including hen age, genetics, housing systems, and activity levels [73].

Tibial morphology and breaking strength in the present study showed no significant differences between the AM/PM treatment and the control group. These findings are partially supported by Molnár et al. [77], who also observed that AM/PM feeding had no significant effect on tibia-breaking strength. However, the observed lower ash content in the AM/PM treatment compared to the control in the current study contrasts with the findings of Molnár et al. [77] and Moss et al. [78], who reported higher leg bone ash in AM/PM treatments. This discrepancy suggests that the AM/PM treatment in the present study resulted in lower mineral deposition within the bone despite hens showing numerically higher serum Ca levels. One potential reason for this could be the reduced intake of Ca-and-vitamin D-supplemented PM water in the AM/PM treatment. It is interesting that the other bone parameters, like breaking strength, remain unaffected despite a lower bone ash content in the hens of AM/PM treatment compared to the control hens. This observation may be attributed to several factors. Firstly, bone ash content reflects mineral density but is not the only factor that preserves bone strength or structural integrity [79]. It is possible that the hens maintaining an adequate bone architecture and collagen matrix exhibit better breaking strength [80,81]. Additionally, the timing of Ca supplementation with vitamin D in the AM/PM group might have influenced Ca utilization without significantly affecting bone strength. Another possibility is that other compensatory mechanisms, such as improved Ca absorption efficiency or redistribution of minerals, maintained bone function. Additional dietary vitamin D supplements could also play a role. Further investigation into Ca metabolism and bone-remodeling processes would help clarify these findings.

The present study’s results on hens’ phenotypic welfare traits revealed that hens in the AM/PM treatment exhibited reduced feather damage in the chest and belly areas compared to those in the control group at 65 WOA. Additionally, hens in the AM/PM treatment tended to have better feather coverage in the neck and wing regions. These observations suggest that hens of the AM/PM treatment may have reduced aggression or feather-pecking behavior, contributing to improved welfare compared to the control diet. This conclusion aligns with the findings of Moss et al. [78], who reported that AM/PM hens were significantly less inclined toward feather pecking than control hens. The potential mechanism behind this observation could be that the AM/PM diet more accurately meets the nutrient requirements, thereby alleviating any nutritional stress and ultimately reducing pecking behavior. However, this finding was observed at only one time point (week 10 of the trial) and was limited to two specific body areas of the hens. Therefore, further investigations are needed to validate this hypothesis in future studies.

## 5. Conclusions

Overall, the performance of hens in the AM/PM treatment that offered Ca and vitamin D through afternoon water did not significantly differ from that of the control group. However, several benefits were observed, including significantly reduced water consumption without impacting overall production, improved energy digestibility and vitamin D levels, and reduced feather damage. This innovative feeding approach shows promise for the practical implementation on farms using existing water-dosing systems.

## Figures and Tables

**Table 1:** Composition of dietary treatments.

Ingredient (%)	Control	AM/PM
Wheat	50.9	52.2
Barley	10.0	10.0
Soybean meal	12.9	12.7
Rapeseed meal	10.0	10.0
Vegetable oil	3.7	3.6
^1^ Limestone 38 Flour	5.5	4.9
^2^ Limestone 38 Grit	5.5	4.9
Salt	0.159	0.157
Monocalcium phosphate	0.315	0.313
Sodium bicarbonate	0.238	0.240
L-lysine HCl	0.058	0.061
DL-methionine	0.139	0.136
L-threonine	0.009	0.010
Choline chloride 60%	0.028	0.027
^3^ Vitamin and mineral layer premix	0.100	0.100
^4^ Pigment red (Jabiru Red)	0.004	0.004
^5^ Pigment yellow (Jabiru Yellow)	0.003	0.003
^6^ Xylanase (Axtra XB)	0.050	0.050
^7^ Phytase (Axtra Phy)	0.006	0.006
Titanum dioxide (TiO_2_)	0.500	0.500

^1^ Limestone 38 Flour: Attunga AGLime, Ca 38.4% (96% as calcium carbonate), neutralizing value 97.5%, fineness minimum 95% (passing 0.71 mm sieve) and 55% fines (passing 0.25 mm sieve), Graymont (Australia) Pty. Ltd. (Braeside, Australia); ^2^ Limestone 38 Grit: Poultry Grit, minimum Ca as calcium carbonate 39%, minimum neutralizing value 98%, Sizing 3.5 mm to 1000 microns, Australian Agricultural Mineral (AAM); ^3^ Vitamin–mineral premix included the following per kilogram of diet: 10,000 IU of vitamin A, 3000 IU of vitamin D, 20 mg of vitamin E, 3 mg of vitamin K, 35 mg of nicotinic acid (niacin), 12 mg of pantothenic acid, 1 mg of folic acid, 6 mg of riboflavin (B2), 0.02 mg of cyanocobalamin (B12), 0.1 mg of biotin, 5 mg of pyridoxine (B6), 2 mg of thiamine (B1), 8 mg of copper as copper sulphate pentahydrate, 0.2 mg of cobalt as cobalt sulphate 21%, 0.5 mg of molybdenum as sodium molybdate, 1 mg of iodine as potassium iodide 68%, 0.3 mg of selenium as selenium 2%, 60 mg of iron as iron sulphate 30%, 60 mg of zinc as zinc sulphate 35%, 90 mg of manganese as manganous oxide 60%, and 20 mg of antioxidant. ^4^ Pigment red (Jabiru red): Canthaxanthin 10%, Guangzhou Juyuan Bio-Chem Co., Ltd. (Guangzhou, China); ^5^ Pigment yellow (Jabiru yellow): Apocarotenoic acid ethyl ester 10%, Guangzhou Juyuan Bio-Chem Co., Ltd.; ^6^ Xylanase: Axtra XB TPT 201, Danisco Animal Nutrition (IFF); ^7^ Phytase: AxtraPHY Gold, Danisco Animal Nutrition (IFF).

**Table 2:** Nutritional composition of dietary treatments.

Calculated Values (%)	Control	AM/PM
Dry matter	89.99	89.88
^1,2^ AME (Kcal/kg)	2777	2808
Crude protein	16.63	16.66
Crude fiber	3.51	3.53
Crude fat	5.24	5.20
Ash	13.09	12.07
Dig ^3,4^ lysine	0.730	0.730
Dig methionine	0.384	0.382
Dig methionine+cystine	0.660	0.660
Dig cysteine	0.274	0.276
Dig threonine	0.510	0.510
Dig tryptophan	0.197	0.197
Dig arginine	0.859	0.858
Dig serine	0.597	0.599
Dig histidine	0.351	0.351
Dig isoleucine	0.590	0.590
Dig leucine	1.016	1.017
Dig phenylalanine	0.674	0.675
Dig tyrosine	0.537	0.538
Dig valine	0.687	0.688
Calcium (Ca)	4.50	4.10
Available phosphorus (P)	0.38	0.38
Sodium	0.18	0.18
Chloride	0.18	0.18
Potassium	0.67	0.67
Linoleic acid	1.20	1.20
Choline (mg/kg)	1800	1800
Dietary electrolyte balance (mEq/kg)	199.09	199.48
Analyzed values (%)		
Dry matter	90.76	90.05
^5^ GE (MJ/kg)	14.63	15.29
Crude protein	16.11	16.43
Ca	5.53	4.5
P	0.48	0.48

^1^ AME = Apparent metabolizable energy. ^2^ Diet formulation to keep values the exact same is difficult in this instance as the PM diet, in particular, needs to be very low in nutrients. Thus, the PM diet would benefit from having a new low-density ingredient added to allow for ease of formulation. However, as this is a scientific experiment, we did not want to introduce a new ingredient as this may confound the design. Thus, we came as close as we could (1% difference) to the control and nutrient requirement while utilizing the same ingredients, ^3^ Dig = Digestible, ^4^ Digestible amino acid coefficients for raw ingredients were determined by near-infrared spectroscopy (Foss NIR 6500, Denmark) standardized with Evonik AMINONIR^®^ Advanced calibration, ^5^ GE = Gross energy.

**Table 3:** Effect of Ca and vitamin D supplementation in evening water on laying performance of hens from week 1–10 of this study.

Weeks	Parameters	Control	AM/PM	SEM	*p*-Value
1–5	Average Egg Weight (g)	62.36	62.12	0.33	0.624
	Egg Mass (g)	58.44	58.80	0.59	0.681
	Hen Day Egg Production (%)	93.72	94.49	0.86	0.549
	^1^ FCR	2.613	2.459	0.070	0.149
6–10	Average Egg Weight (g)	61.83	61.82	0.26	0.983
	Egg Mass (g)	54.78	55.16	0.68	0.694
	Hen Day Egg Production (%)	88.61	89.25	1.05	0.672
	FCR	2.846	2.703	0.043	0.075
1–10	Average Egg Weight (g)	62.09	61.97	0.29	0.773
	Egg Mass (g)	56.61	56.67	0.66	0.949
	Hen Day Egg Production (%)	91.16	91.44	0.95	0.845
	FCR	2.692	2.615	0.074	0.472

^1^ FCR = Feed conversion ratio.

**Table 4:** Effect of Ca and vitamin D supplementation in evening water on average feed and water intake of hens from week 1–10 of this study.

Weeks	Parameters	Control	AM/PM	SEM	*p*-Value
1–5	Feed intake (g/bird/day)	152.60	142.98	3.91	0.112
	Water intake (ml/bird/day)	194.24 ^a^	167.51 ^b^	1.44	<0.001
6–10	Feed intake (g/bird/day)	152.49	147.64	2.85	0.246
	Water intake (ml/bird/day)	196.22 ^a^	174.02 ^b^	2.71	0.009
1–10	Feed intake (g/bird/day)	152.55	144.41	3.01	0.083
	Water intake (ml/bird/day)	196.85 ^a^	171.23 ^b^	1.36	<0.001

^a,b^ Means in the same row not sharing a common suffix are significantly different at the 5% level of probability.

**Table 5:** Effect of Ca and vitamin D supplementation in evening water on external and internal quality of hen eggs at weeks 5 and 10 of this study.

Week	Parameters	Control	AM/PM	SEM	*p*-Value
Week 5	Eggshell reflectivity	28.58	29.93	0.53	0.099
	Egg shape index	0.759 ^b^	0.769 ^a^	0.00	0.021
	Yolk weight (g)	16.04	15.84	0.20	0.517
	Shell weight (g)	6.01	6.11	0.04	0.111
	Shell thickness (mm)	0.43	0.43	0.00	0.724
	Egg weight (g)	62.94	62.51	0.52	0.577
	Albumen height (mm)	7.96	8.60	0.26	0.116
	Yolk color	11.13	10.85	0.36	0.598
	Haugh unit	86.99	90.39	1.48	0.136
	Eggshell breaking strength (kgf)	4.00	4.11	0.11	0.489
	Yolk height (mm)	21.03	21.35	0.19	0.258
	Yolk diameter (mm)	40.23	40.12	0.32	0.843
	Yolk index	0.52	0.52	0.01	0.979
Week 10	Eggshell reflectivity	28.63	28.60	0.41	0.969
	Egg shape index	0.757	0.760	0.00	0.173
	Yolk weight (g)	16.04	16.01	0.14	0.868
	Shell weight (g)	6.10	6.06	0.05	0.700
	Shell thickness (mm)	0.43	0.43	0.00	0.488
	Egg weight (g)	62.54	62.19	0.53	0.687
	Albumen height (mm)	8.21	8.28	0.30	0.869
	Yolk color	12.49	12.84	0.24	0.320
	Haugh unit	88.03	88.94	1.90	0.737
	Eggshell breaking strength (kgf)	3.77	3.77	0.09	0.989
	Yolk height (mm)	21.46	21.20	0.09	0.076
	Yolk diameter (mm)	39.62	39.66	0.53	0.959
	Yolk index	0.55	0.54	0.01	0.432

^a,b^ Means in the same row not sharing a common suffix are significantly different at the 5% level of probability.

**Table 6:** Effect of Ca and vitamin D supplementation in evening water on hen weight, CV% of hen weight, egg weight, CV% of egg weight at weeks 5 and 10 of this study.

Week	Parameters	Control	AM/PM	SEM	*p*-Value
5	Hen weight (g/hen)	2194	2172	15.03	0.307
	CV% of hen weight	8.04	9.44	0.46	0.067
	Egg weight (g/egg)	61.86	61.24	0.39	0.277
	CV% of egg weight	6.98	7.91	0.45	0.178
10	Hen weight (g/hen)	2199	2173	15.05	0.227
	CV% of hen weight	8.36	9.88	0.54	0.071
	Egg weight (g/egg)	62.54	62.19	0.53	0.688
	CV% of egg weight	5.25	5.93	0.56	0.423

**Table 7:** Effect of Ca and vitamin D supplementation in evening water on the digestibility of nutrients of hens at week 10 of this study.

Parameters ^1^	Control	AM/PM	SEM	*p*-Value
IDE as DM	9.06 ^b^	10.00 ^a^	0.241	0.014
IDEC as DM	0.56	0.59	0.015	0.212
IDNC as DM	0.71	0.73	0.011	0.368

^1^ IDE: ileal digestible energy, IDEC: ileal digestible energy coefficient, IDNC: ileal digestible nitrogen coefficient, DM: Dry matter; ^a,b^ Means in the same row not sharing a common suffix are significantly different at the 5% level of probability.

**Table 8:** Effect of Ca and vitamin D supplementation in evening water on serum Ca and vitamin D (Vit-D) levels of hens at week 10 of this study.

Parameters	Control	AM/PM	SEM	*p*-Value
Serum Ca (mg/dL)	29.74	31.99	0.85	0.089
Serum Vit-D (ng/mL)	22.64 ^b^	27.59 ^a^	1.21	0.014

^a,b^ Means in the same row not sharing a common suffix are significantly different at the 5% level of probability.

**Table 9:** Effect of Ca and vitamin D supplementation in evening water on average kidney weight (g/kg bw), liver weight (g/kg bw) and keel bone damage score of hens from different treatments at week 10 of this study.

Parameters	Control	AM/PM	SEM	*p*-Value
Kidney weight (g/kg bw)	4.11	4.04	0.21	0.814
Liver weight (g/kg bw)	19.44	18.43	0.59	0.248
Keel bone damage score *	0.81	0.58	0.15	0.299

* For keel bone damage score, 0, 1, and 2 indicate normal/healthy keel, hairline fracture and broken keel, respectively.

**Table 10:** Effect of Ca and vitamin D supplementation in evening water on tibia characteristics of hens at week 10 of this study.

Parameters	Control	AM/PM	SEM	*p*-Value
Fresh weight (g)	12.83	13.07	0.16	0.311
Air-dry weight (g)	9.76	10.04	0.16	0.236
Length (mm)	122.66	123.65	0.50	0.209
Width (mm)	7.06	7.14	0.08	0.553
Seedor Index (SI)	79.83	80.75	1.24	0.606
Breaking Strength (Nw)	238.17	218.92	12.95	0.309
Ash as-is (%)	42.30 ^a^	39.73 ^b^	0.73	0.024

^a,b^ Means in the same row not sharing a common suffix are significantly different at the 5% level of probability.

**Table 11:** Effect of Ca and vitamin D supplementation in evening water on hen welfare at weeks 5 and 10 of this study.

Week	Parameters	Control	AM/PM	SEM	*p*-Value
Week 5	Comb wound	0.21	0.23	0.05	0.825
	Body condition score	1.03	0.92	0.08	0.371
	Neck feather	0.53	0.49	0.10	0.793
	Chest feather	0.25	0.12	0.08	0.302
	Back feather	0.06	0.08	0.02	0.473
	Wing feather	0.05	0.03	0.02	0.457
	Belly feather	0.74	0.78	0.15	0.850
Week 10	Comb wound	0.28	0.44	0.07	0.129
	Body condition score	0.73	0.81	0.07	0.380
	Neck feather	0.75	0.65	0.04	0.092
	Chest feather	1.16 ^a^	0.91 ^b^	0.06	0.028
	Back feather	0.15	0.08	0.03	0.200
	Wing feather	0.09	0.04	0.02	0.078
	Belly feather	1.55 ^a^	1.34 ^b^	0.06	0.021

^a,b^ Means in the same row not sharing a common suffix are significantly different at the 5% level of probability. For feather score (neck, chest, back, wings, tail, and belly), 1, 2, 3, and 4 indicate severe, moderate, mild, and no damage, respectively. For body condition score, 0, 1, and 2 indicate fat, normal, and skinny, respectively.

## Data Availability

The research data supporting this study will be shared upon a reasonable request made to the corresponding author. The data are not publicly available due to privacy.
